# Supplementary material for: Protocol on establishing a national disease registry–Swiss Pediatric Inflammatory Brain Disease Registry
Source: PLoS One. 2024 Apr 16;19(4):e0290908. doi: 10.1371/journal.pone.0290908 (PMC11020937; doi:10.1371/journal.pone.0290908)
Supplement: Attachment — Submitted filename: Reviewer.docx [file pone.0290908.s001.docx]

Reviewer’s comments

Line number 32,33:

1.Are you referring to clinical trials here?

2.For whom the studies will be accessible to and how will you make them accessible?

Line number 89-90:

Is there an estimate how many patients on an average from these French and Italian registries who participated and benefitted from a new clinical trial.It would be nice to know.

Line number 91,92:

Which registries will you link with?European only or other parts of the world.Are there any other Pediatric inflammatory brain disease registries in the world.

Line number 101,102:

Research studies or trials for new drugs?

Line number 107:

How will you ensure that previously diagnosed patients(from 2005 onwards) are not missed as diagnoses of Pediatric inflammatory brain diseases evolve over time.

Line number 112:

How will the PI shortlist eligible study participants from screening list.

Line number 113,114,115:

What will be the procedure of informed consent for such patients whom you are unable of contact/not regularly visiting clinics and you are sending letters to.

Line number 117,118:

This recruitment includes new patients or older ones from 2005 onwards.

Line number 132,133:

How will you sustain the availability of logistics/manpower for continuation of this process at ISPM for long term as the project has no end point.

Line number 213,214:

1.are these mostly newly diagnosed patients or older ones?

2.Are Eligible participants shortlisted from screened participants list?

Line number 231,232:

This way the older cohort may be missed as definitive diagnostic tests may not have been available previously which are now.

Line number 233,234,235:

1.How does centralizing the data collection help in recruiting missing data?

2.Will the executive office member will collect missing data of patients?
