# Supplementary material for: Protocol on establishing a national disease registry–Swiss Pediatric Inflammatory Brain Disease Registry
Source: PLoS One. 2024 Apr 16;19(4):e0290908. doi: 10.1371/journal.pone.0290908 (PMC11020937; doi:10.1371/journal.pone.0290908)
Supplement: Attachment — Submitted filename: 240126_Response to Reviewers.docx [file pone.0290908.s002.docx]

Lorena Freya Hulliger, MSc

University of Bern, Institute of Social and Preventive Medicine

Mittelstrasse 43, Bern, Switzerland

Bern, 1/26/2024

Dear Dr. Emily Chenette,

Dear Editors of PLOS ONE,

Dear Reviewers

Thank you for your careful review of our manuscript. On the following pages we have tried to answer each point raised during the review to the best of our ability.

Additionally, upon your request we have included the below statement in the cover letter and removed all funding information from the manuscript. Please update the online submission form on our behalf with the following funding statement:

*“All funding was awarded to S.B. The project is funded by the following organizations: Swiss Multiple Sclerosis Society (www.multiplesklerose.ch), Roche Pharma (Switzerland) Ltd (award number: 20 f 9-GR-0074; www.roche.ch), Novartis Pharma Schweiz AG (award number: P3 C11912780147; www.novartis.com), and Sanofi-Aventis (Schweiz) SA (www.sanofi.ch). The funders had no role in the study design, data collection and analysis, decision to publish, or preparation of the manuscript.”*

In the hope that we have provided you with a satisfactory response, we remain at your disposal.

With kind regards,

Lorena Hulliger, corresponding author

# PLOS ONE Journal Requirements

## Requirement 1.:

*Please ensure that your manuscript meets PLOS ONE's style requirements, including those for file naming. The PLOS ONE style templates can be found at https://journals.plos.org/plosone/s/file?id=wjVg/PLOSOne_formatting_sample_main_body.pdf and* [*https://journals.plos.org/plosone/s/file?id=ba62/PLOSOne_formatting_sample_title_authors_affiliations.pdf*](https://journals.plos.org/plosone/s/file?id=ba62/PLOSOne_formatting_sample_title_authors_affiliations.pdf)

## Answer:

The manuscript has been changed to meet PLOS ONE’s style requirements.

## Requirement 2.:

*We note that the grant information you provided in the ‘Funding Information’ and ‘Financial Disclosure’ sections do not match. When you resubmit, please ensure that you provide the correct grant numbers for the awards you received for your study in the ‘Funding Information’ section.*

## Answer:

The sections ‘Funding Information and ‘Financial Disclosure’ have been changed and are now identical. Please note, that the grant numbers have been added when possible – some grants/donations had no official grant numbers.

## Requirement 3.:

*Thank you for stating the following in your Competing Interests section: "None of the authors have any potentially competing interest to declare."*

*Please complete your Competing Interests on the online submission form to state any Competing Interests. If you have no competing interests, please state "The authors have declared that no competing interests exist.", as detailed online in our guide for authors at* [*http://journals.plos.org/plosone/s/submit-now*](http://journals.plos.org/plosone/s/submit-now)*. This information should be included in your cover letter; we will change the online submission form on your behalf.*

## Answer:

The required statement "The authors have declared that no competing interests exist." has been added to the ‘Competing Interests’ section on the online submission form. The statement has also been included in the cover letter.

## Requirement 4.:

*PLOS requires an ORCID iD for the corresponding author in Editorial Manager on papers submitted after December 6th, 2016. Please ensure that you have an ORCID iD and that it is validated in Editorial Manager. To do this, go to ‘Update my Information’ (in the upper left-hand corner of the main menu), and click on the Fetch/Validate link next to the ORCID field. This will take you to the ORCID site and allow you to create a new iD or authenticate a pre-existing iD in Editorial Manager. Please see the following video for instructions on linking an ORCID iD to your Editorial Manager account:* [*https://www.youtube.com/watch?v=_xcclfuvtxQ*](https://www.youtube.com/watch?v=_xcclfuvtxQ)

## Answer:

A new ORCID iD has been created and validated in Editorial Manager.

## Requirement 5.:

*Your ethics statement should only appear in the Methods section of your manuscript. If your ethics statement is written in any section besides the Methods, please delete it from any other section.*

## Answer:

The ethics statement has been deleted from other sections and appears exclusively in the Methods section of the manuscript.

## Requirement 6.:

*We note that Figure 1 in your submission contain copyrighted image. All PLOS content is published under the Creative Commons Attribution License (CC BY 4.0), which means that the manuscript, images, and Supporting Information files will be freely available online, and any third party is permitted to access, download, copy, distribute, and use these materials in any way, even commercially, with proper attribution. For more information, see our copyright guidelines: http://journals.plos.org/plosone/s/licenses-and-copyright.*

*We require you to either (1) present written permission from the copyright holder to publish these figures specifically under the CC BY 4.0 license, or (2) remove the figures from your submission:*

*a. You may seek permission from the original copyright holder of Figure 1 to publish the content specifically under the CC BY 4.0 license.*

*We recommend that you contact the original copyright holder with the Content Permission Form (http://journals.plos.org/plosone/s/file?id=7c09/content-permission-form.pdf) and the following text:*

*“I request permission for the open-access journal PLOS ONE to publish XXX under the Creative Commons Attribution License (CCAL) CC BY 4.0 (http://creativecommons.org/licenses/by/4.0/). Please be aware that this license allows unrestricted use and distribution, even commercially, by third parties. Please reply and provide explicit written permission to publish XXX under a CC BY license and complete the attached form.”*

*Please upload the completed Content Permission Form or other proof of granted permissions as an ""Other"" file with your submission.*

*In the figure caption of the copyrighted figure, please include the following text: “Reprinted from [ref] under a CC BY license, with permission from [name of publisher], original copyright [original copyright year].”*

*b. If you are unable to obtain permission from the original copyright holder to publish these figures under the CC BY 4.0 license or if the copyright holder’s requirements are incompatible with the CC BY 4.0 license, please either i) remove the figure or ii) supply a replacement figure that complies with the CC BY 4.0 license. Please check copyright information on all replacement figures and update the figure caption with source information. If applicable, please specify in the figure caption text when a figure is similar but not identical to the original image and is therefore for illustrative purposes only.*

## Answer:

Figure 1 has been removed from the submission.

## Requirement 7.:

*Please review your reference list to ensure that it is complete and correct. If you have cited papers that have been retracted, please include the rationale for doing so in the manuscript text, or remove these references and replace them with relevant current references. Any changes to the reference list should be mentioned in the rebuttal letter that accompanies your revised manuscript. If you need to cite a retracted article, indicate the article’s retracted status in the References list and also include a citation and full reference for the retraction notice.*

## Answer:

The reference list has been reviewed and was found to be complete and correct.

# Reviewers’ comments

## Comment 1.:

*Does the manuscript provide a valid rationale for the proposed study, with clearly identified and justified research questions?*

*Reviewer #1: Yes*

*Reviewer #2: Yes*

## Answer:

Thank you for the positive review.

## Comment 2.:

*Is the protocol technically sound and planned in a manner that will lead to a meaningful outcome and allow testing the stated hypotheses?*

*The manuscript should describe the methods in sufficient detail to prevent undisclosed flexibility in the experimental procedure or analysis pipeline, including sufficient outcome-neutral conditions (e.g. necessary controls, absence of floor or ceiling effects) to test the proposed hypotheses and a statistical power analysis where applicable. As there may be aspects of the methodology and analysis which can only be refined once the work is undertaken, authors should outline potential assumptions and explicitly describe what aspects of the proposed analyses, if any, are exploratory.*

*Reviewer #1: Yes*

*Reviewer #2: Yes*

## Answer:

Thank you for the positive review.

## Comment 3.:

*Is the methodology feasible and described in sufficient detail to allow the work to be replicable?*

*Descriptions of methods and materials in the protocol should be reported in sufficient detail for another researcher to reproduce all experiments and analyses. The protocol should describe the appropriate controls, sample size calculations, and replication needed to ensure that the data are robust and reproducible.*

*Reviewer #1: Yes*

*Reviewer #2: Yes*

## Answer:

Thank you for the positive review.

## Comment 4.:

*Have the authors described where all data underlying the findings will be made available when the study is complete?*

*The PLOS Data policy requires authors to make all data underlying the findings described in their manuscript fully available without restriction, with rare exception, at the time of publication. The data should be provided as part of the manuscript or its supporting information, or deposited to a public repository. For example, in addition to summary statistics, the data points behind means, medians and variance measures should be available. If there are restrictions on publicly sharing data—e.g. participant privacy or use of data from a third party—those must be specified.*

*Reviewer #1: Yes*

*Reviewer #2: No*

## Answer:

Thank you for raising this point.

Since the manuscript does not contain any data besides the number of recruited participants, we feel, that regarding this publication we have met the PLOS Data policy requirement of “all data underlying the findings described in their manuscript fully available without restriction”.

Regarding subsequent publications, we will make as much data available as possible without compromising participant privacy.

## Comment 5.:

*Is the manuscript presented in an intelligible fashion and written in standard English?*

*PLOS ONE does not copyedit accepted manuscripts, so the language in submitted articles must be clear, correct, and unambiguous. Any typographical or grammatical errors should be corrected at revision, so please note any specific errors here.*

*Reviewer #1: Yes*

*Reviewer #2: Yes*

## Answer:

Thank you for the positive review.

## Comment 6.:

*Review Comments to the Author*

*Please use the space provided to explain your answers to the questions above and, if applicable, provide comments about issues authors must address before this protocol can be accepted for publication. You may also include additional comments for the author, including concerns about research or publication ethics.*

*You may also provide optional suggestions and comments to authors that they might find helpful in planning their study.*

*(Please upload your review as an attachment if it exceeds 20,000 characters)*

*Reviewer #1: I wanted to inquire about some of the process flow regarding recruitment of participants in study which i have mentioned in my comments in word file.*

*Reviewer #2: The authors may clarify under the Data Availability section whether all data underlying the findings will be made available when the study is complete.*

*Furthermore, the study period may also be clarified.*

## Answer:

### Answer to the comments of Reviewer 1:

- *Line number 32,33:*

*1.Are you referring to clinical trials here?*

The type of trial is unrestricted. But yes, our protocol states, that we can contact the patients/families in the registry for recruitment purposes. This way we can ask our patients if they would like to participate in e.g. international trials that would be of interest to them.

*2.For whom the studies will be accessible to and how will you make them accessible?*

For all patients/families that have given their informed consent to participate in the registry and who meet the inclusion criteria of the study. E.g., if we are contacted by an institution conducting a study to research paediatric MS (with a formal ethics committee approval), we can collaborate with them and send their informed consent forms and invitation for participation to our patients.

- *Line number 89-90:*

*Is there an estimate how many patients on an average from these French and Italian registries who participated and benefitted from a new clinical trial.It would be nice to know.*

We do not have an estimate of how many Italian/French patients have been recruited to participate in clinical trials through the centralized registries.

- *Line number 91,92:*

*Which registries will you link with? European only or other parts of the world. Are there any other Pediatric inflammatory brain disease registries in the world.*

We are working on a collaboration with the Dutch registry for demyelinating diseases in children. We intend to link with registries all over the world. But this will take time and a lot of work. There are other paediatric inflammatory brain disease registries such as the Canadian Brain Works. However, the registries sometimes include only one IBrainD, such as paediatric onset MS.

- *Line number 101,102:*

*Research studies or trials for new drugs?*

Both, if possible.

- *Line number 107:*

*How will you ensure that previously diagnosed patients (from 2005 onwards) are not missed as diagnoses of Pediatric inflammatory brain diseases evolve over time.*

The possibility that we will miss patients that have been diagnosed as far back as 2005 is high.

Our priority however, are the patients that have been diagnosed more recently and are therefore still in paediatric care.

We have also already seen a shift of the “diagnoses mix” over time. As an example: the proportion of MOG-AD diagnoses has increased manyfold over the last three years.

We will disclose this issue and discuss it upon the publication of our first epidemiological data. Thank you very much for raising this point.

- *Line number 112:*

*How will the PI shortlist eligible study participants from screening list.*

The PIs of the centres have been trained by the Executive Office to recognise the patients meeting the inclusion criteria. Additionally, the Executive Office checks all patient who consented for suitability.

- *Line number 113,114,115:*

*What will be the procedure of informed consent for such patients whom you are unable of contact/not regularly visiting clinics and you are sending letters to.*

We send them an invitation to participate in the registry together with the information letter. If they agree to participation, we may contact them by telephone to explain the proceedings of the registry. If they do not reply after two invitations, we only collect the minimal dataset and mark them as non-responders.

- *Line number 117,118:*

*This recruitment includes new patients or older ones from 2005 onwards.*

We also recruit patients who have been diagnosed as far back as 2005.

- *Line number 132,133:*

*How will you sustain the availability of logistics/manpower for continuation of this process at ISPM for long term as the project has no end point.*

This is one of our greatest challenges since we are third party funded. Much of our work at ISPM is put into looking for appropriate funders and writing grants. We however have a close collaboration with the Swiss-MS society who is very invested in the project. Furthermore, the Steering Board of the registry supports the acquisition of funds.

- *Line number 213,214:*

*1.are these mostly newly diagnosed patients or older ones?*

This depends on the definition of “newly diagnosed”. We found that we have about 20-30 new cases per year. This means, that most of the patients have been diagnosed before the registry has started recruitment.

*2.Are Eligible participants shortlisted from screened participants list?*

Yes, but the PIs compile the screening lists themselves and are also responsible for the shortlisting.

- *Line number 231,232:*

*This way the older cohort may be missed as definitive diagnostic tests may not have been available previously which are now.*

This is indeed a pertinent risk. We will try to minimize the risk as far as possible by checking the diagnostic work-up that has been done at the time of diagnosis (these variables are included in the registry database). In a publication on first epidemiological data, we will discuss this issue and possibly add it as a limitation.

- *Line number 233,234,235:*

*1.How does centralizing the data collection help in recruiting missing data?*

The collection of data is essentially done by one person. This allows the implementation of a standardized procedure during data collection. If e.g. a patient changes study centre without notifying the registry, the DM who follows all the patients will notice the change and be able to request the data from both treating centres.

*2.Will the executive office member will collect missing data of patients?*

If we have a data gap, we first ask the corresponding PI to either send us the missing data if available or provide a statement to their best knowledge. Sometimes however there is a “real” data gap. Where a patient for example left the country for a while, continued the treatment abroad, and then came back. In this case we will try to find a way to extrapolate/impute the missing values and disclose the procedure.

### Answer to the comments of Reviewer 2:

Please see our answer to comment 4.

On the topic of the study period, we are unable to further clarify. The study is set-up as a registry (which is intended to continuously enrol new patients and collect structural data) without a clear end date. Any nested projects, however will have a start and an end date that will be provided in the corresponding publication.

## Comment 7:

*PLOS authors have the option to publish the peer review history of their article (what does this mean?). If published, this will include your full peer review and any attached files.*

*If you choose “no”, your identity will remain anonymous but your review may still be made public.*

*Do you want your identity to be public for this peer review? For information about this choice, including consent withdrawal, please see our Privacy Policy.*

*Reviewer #1: Yes: MUZNA ARIF*

*Reviewer #2: No*

## Answer:

Thank you both again for your careful review and valuable input.
